# Correction: Sarmadi et al. Attention Horizon as a Predictor for the Fuel Consumption Rate of Drivers. *Sensors* **2022**, *22*, 2301

**DOI:** 10.3390/s25154608

**Published:** 2025-07-25

**Authors:** Hamid Sarmadi, Sławomir Nowaczyk, Rune Prytz, Miguel Simão

**Affiliations:** 1 Center for Applied Intelligent Systems Research, Halmstad University, 302 50 Halmstad, Sweden; slawomir.nowaczyk@hh.se; 2 Stratio Company, 1050-127 Lisbon, Portugal

## Error in Algorithms 1 and 2

Algorithms 1 and 2 have been updated, with the new, correct versions shown below. The lines that needed to be corrected from the original paper [1] are annotated with dashed-line boxes. In the boxes below, you can see the corrections.
**Algorithm 1 **Finding the set of paths before stopping at red traffic lights during a day for a vehicle**Procedure** FindPaths(C, S, T)    *SP* ← { *i* | *s_i_* ∈ *S* ∧ *s_i_* < 1 }// All point indices with slow vehicle speed    *P*←∅// *P*: The set of extracted traffic light paths    ***For**** i* ∈ *SP*        **If **∄ (*R*, *D*) ∈ *P* such that *i* ∈ *R*            *t* ← *T*(*c_i_*)// *t*: coordinates of the closest traffic light            *j* ← *i* + 1            **While*** j* ≤ *N* ∧ (*dist*(*c_j_*, *t*) < *dist*(*c_j_*_−__1_, *t*) ∧ *dist*(*c_j_,* *t*) < 50)// Distance unit is meters                *j* ← *j* + 1            **End While**            **If** *dist*(*c_j_₋_*1*_*, *t*) < 5//Distance unit is meters                **While** *j* > 1 ∧ (*j*−1 ∈ *SP* ∨ *j* ∉ *SP*)//Go back to the last slowdown point                    *j *← *j* − 1                **End While**                *P* ← *P* ∪ CreatePath(*C*, *j*, *t*, *P*)                **Break**            **End If**        **End If**    **End For**    **Return** *P***End Procedure**
**Algorithm 2 **Creating a path given its closest point to the traffic light**Procedure** CreatePath(C, j, t, P)    *k* ← 1    *d_k_* ← 0// *d_k_*: Traversed distance to the stopping point at the path’s *k*-th index    *r_k_* ← *j*// *r_k_*: The data point index at the path’s *k*-th index    **While** *d_k_* < *L_p_* ∧ *r_k_* > 1 ∧ [∄ (*R*, *D*) ∈* P* such that *r_k_*_−__1_ ∈ *R*]        *k* ← *k* + 1        *r_k_* ← *r_k_*_−__1_ − 1        *d_k_* ← *d_k_*_−1_ + *dist*($c_{r_{k}},c_{r_{k-1}}$)    **End While**    *R* ← ⟨*r_*1*_*, *r_*2*_*, ..., *r_k_*⟩    *D* ← ⟨*d_*1*_*, *d_*2*_*, ..., *d_k_*⟩    **If** *d_k_* ≥ *L_p_*// If the path’s traversed distance is greater than *L_p_*        **Return **{(*R*, *D*)}    **Else**        **Return **∅    **End If****End Procedure**

The authors state that the scientific conclusions are unaffected. This correction was approved by the Academic Editor. The original publication has also been updated.

## References

[1] Sarmadi H., Nowaczyk S., Prytz R., Simão M. (2022). Attention Horizon as a Predictor for the Fuel Consumption Rate of Drivers. Sensors.

